# The Mechanotransduction Channel and Organic Cation Transporter Are Critical for Cisplatin Ototoxicity in Murine Hair Cells

**DOI:** 10.3389/fnmol.2022.835448

**Published:** 2022-02-10

**Authors:** Jinan Li, Chang Liu, Samuel Kaefer, Mariam Youssef, Bo Zhao

**Affiliations:** Department of Otolaryngology-Head and Neck Surgery, Indiana University School of Medicine, Indianapolis, IN, United States

**Keywords:** cisplatin, ototoxicity, mechanotransduction, TMIE, organic cation transporter

## Abstract

Cisplatin is one of the most widely used chemotherapeutic drugs across the world. However, the serious ototoxic effects, leading to permanent hair cell death and hearing loss, significantly limit the utility of cisplatin. In zebrafish, the functional mechanotransduction channel is required for cisplatin ototoxicity. However, it is still unclear the extent to which the mechanotransduction channel is involved in cisplatin uptake and ototoxicity in mammalian hair cells. Herein, we show that genetically disrupting mechanotransduction in mouse partially protects hair cells from cisplatin-induced hair cell death. Using a fluorescent-dye conjugated cisplatin, we monitored cisplatin uptake in cochlear explants and found that functional mechanotransduction is required for the uptake of cisplatin in murine hair cells. In addition, cimetidine, an inhibitor of the organic cation transporter, also partially protects hair cells from cisplatin ototoxicity. Notably, the otoprotective effects of cimetidine do not require mechanotransduction. These findings suggest that both the mechanotransduction channel and the organic cation transporter are critical for cisplatin ototoxicity in murine hair cells.

## Introduction

Hair cells, the sensory receptors of auditory system, are susceptible to numerous insults such as noise, ototoxic drugs, trauma and aging. Cisplatin is a chemotherapeutic drug widely used to treat various types of cancers, including testicular, ovarian, bladder, head and neck, lung, and cervical cancers. However, patients treated with cisplatin frequently suffer from nausea, vomiting, fatigue, serious kidney problems, tinnitus and hearing loss. The strong ototoxicity of cisplatin, which leads to the permanent hair cell death and irreversible hearing loss in 22–77% of patients, significantly limits its usage in clinics (Knight et al., 2005; Kushner et al., 2006; Coradini et al., 2007; Sheth et al., 2017; Kros and Steyger, 2019; Meijer et al., 2021).

Over the years, the understanding of the ototoxic mechanisms of cisplatin has increased (Sheth et al., 2017; Hazlitt et al., 2018; Kros and Steyger, 2019; Rybak et al., 2019; Prayuenyong et al., 2021). Systemically administrated cisplatin traffics across the blood–labyrinth barrier, enters hair cells predominantly from their apical surface, and remains in the cochlea for months to years (Chu et al., 2016; Breglio et al., 2017). After entry into hair cells, cisplatin induces an accumulation of platinated DNA and reactive oxygen species (ROS), activation of BRAF/MEK/ERK cellular pathway, and ultimately leads to hair cell damage and permanent hearing loss (Sheth et al., 2017; Kros and Steyger, 2019; Ingersoll et al., 2020; Ramkumar et al., 2021). However, the entry routes of cisplatin into hair cells are still not very clear. In cancer cells, copper transport 1 (CTR1) and organic cation transporter 2 (OCT2) have been revealed to mediate cisplatin uptake (Harrach and Ciarimboli, 2015). In HEI-OC1 cells, an auditory cell line derived from the mouse organ of Corti, as well as in neonatal rat cochlear explants, CTR1 and OCT2 mediate cisplatin-induced ototoxicity, suggesting that the influx of cisplatin into hair cells occurs *via* the above two proteins (Ciarimboli et al., 2010; More et al., 2010; Ding et al., 2011). Notably, mice lacking organic cation transporters are insusceptible to cisplatin ototoxicity (Ciarimboli et al., 2010). However, inhibiting CTR1 or OCT2 does not completely protect hair cells from cisplatin-induced hair cell death (Ding et al., 2011), indicating that there are some other proteins involved in cisplatin uptake and ototoxicity. Interestingly, in the lateral line hair cells of zebrafish, functional mechanotransduction but not CTR1 or OCT2 is required for cisplatin-induced hair cell death (Thomas et al., 2013). Several other studies have also indicated a potential role of the mechanotransduction channel in cisplatin ototoxicity (Waissbluth and Daniel, 2013). ORC-13661, a high-affinity permeant blocker of the mechanotransduction channel, protects murine hair cells from cisplatin ototoxicity (Kitcher et al., 2019). In addition, in chicken hair cells, high doses of cisplatin are able to block mechanotransduction currents (Kimitsuki et al., 1993). However, direct evidence of an essential role of mechanotransduction in cisplatin uptake and ototoxicity in murine hair cells is still missing.

The mechanotransduction machinery of hair cells is localized within the stereocilia that protrude from the apical surface of hair cells. The mechanotransduction machinery in hair cells is formed by several distinctive proteins, including transmembrane channel-like 1 and 2 (TMC1/2), LHFPL tetraspan subfamily member 5 protein (LHFPL5; previously named TMHS), transmembrane inner ear protein (TMIE), calcium and integrin-binding family member 2 (CIB2), and tip link proteins (Protocadherin-15 and Cadherin-23) (Siemens et al., 2004; Sollner et al., 2004; Kazmierczak et al., 2007; Kawashima et al., 2011; Xiong et al., 2012; Zhao et al., 2014; Giese et al., 2017; Pan et al., 2018; Pacentine and Nicolson, 2019; Cunningham et al., 2020). Previous studies have found that TMIE is an essential subunit of the mechanotransduction channel defining its pore and gating properties (Zhao et al., 2014; Cunningham et al., 2020). Notably, murine hair cells that lack TMIE have no mechanotransduction currents (Zhao et al., 2014; Cunningham et al., 2020).

In this study, we compared the murine hair cell death caused by cisplatin in wild-type and TMIE-deficient cochlear explants. Furthermore, using commercially available Texas Red-conjugated cisplatin (TR–cisplatin), we investigated the cisplatin uptake in wild-type and TMIE-deficient hair cells. Since OCT2 has been reported to mediate the toxicity of cisplatin in cancer cells and murine hair cells (Harrach and Ciarimboli, 2015), we studied the otoprotective effects of cimetidine on wild-type and TMIE-deficient hair cells. Our results suggest that both the mechanotransduction channel and OCT2 are critical for cisplatin ototoxicity in murine hair cells.

## Materials and Methods

### Animal Models and Animal Care

*Tmie*^–/–^ mice (MGI: 5784557) have been described previously (Zhao et al., 2014). More than three mice per group, including both male and female mice, were used. All of the animal experiments were carried out in accordance with the National Institutes of Health Guide and were approved by the Institutional Animal Care and Use Committee of Indiana University School of Medicine (IACUC; Protocol #19075).

### Cochlear Explants Culture and TR-Cisplatin Uptake Assay

Cochlear explants were dissected from P3 mice and cultured in DMEM/F12 medium (Cat.# 21041025, Life Technologies Corporation) overnight at 37°C in a 5% CO2 humidified atmosphere. Then, the samples were incubated in DMEM/F12 medium containing various concentrations of cisplatin (Cat.# 232120, Millipore Sigma, dissolved in DMEM/F12) for 2 days at 37°C. Cimetidine (Cat.# AAJ6282506, Fisher Scientific) was also dissolved in DMEM/F12 and added to the cochlear explants prior to adding of cisplatin. To monitor the cisplatin uptake in hair cells, the cochlear explants were incubated in DMEM/F12 medium containing 2 μg/mL of TR–cisplatin (Ursa BioScience) for various amounts of time. Stacked images were then captured using a deconvolution microscope (Leica) with a 63 × objective (HCX APO L63X/0.90 Water). The images were then deconvoluted using the blind deconvolution method.

### Immunostaining

Immunostaining was performed as described previously (Liu et al., 2018). In brief, the cochlear explants were fixed in 4% PFA for 20 min at room temperature and then washed three times for 5 min each in HBSS. The tectorial membrane was then removed and the samples were blocked in blocking buffer (5% bovine serum albumin and 0.5% Triton X-100 in HBSS solution) for 20 min at room temperature. Then, the samples were incubated with primary antibodies diluted in antibody dilution buffer (1% BSA and 0.1% Triton X-100 in HBSS solution) overnight at 4°C. After being washed with HBSS, the samples were incubated with secondary antibodies for 2 h at room temperature. Then, the samples were mounted in ProLong ^®^ Antifade Reagents (Cat.# P36971, Life technologies Corporation). Stacked images were then captured using a deconvolution microscope (Leica) with a 20 × objective (HC PL FLUOTAR 20x/0.55) or a 100 × objective (HCX PL APO 100x/1.40-0.70 OIL). Antibodies used in this study were anti-β2 spectrin (1:200, Cat.# sc-136074, Santa Cruz) and Alexa Fluor 488 goat anti-mouse (1:2,000, Cat.# A11017, Life technologies Corporation).

### Data Analysis

At least three different animals, including both genders, were used for each experiment. The precise numbers, sample size, repetitions, and statistical tests are indicated in the figures and figure legends. The Kolmogorov–Smirnov test was used to determine the normality of data distribution. Then two-way ANOVA followed by Bonferroni *post hoc* test or two-tailed unpaired Student’s *t*-test was used to determine statistical significance (*p < 0.05, **p < 0.01, and ***p < 0.001).

## Results

### Cisplatin Dose-Dependently Kills Wild-Type Murine Hair Cells

**FIGURE 1:**
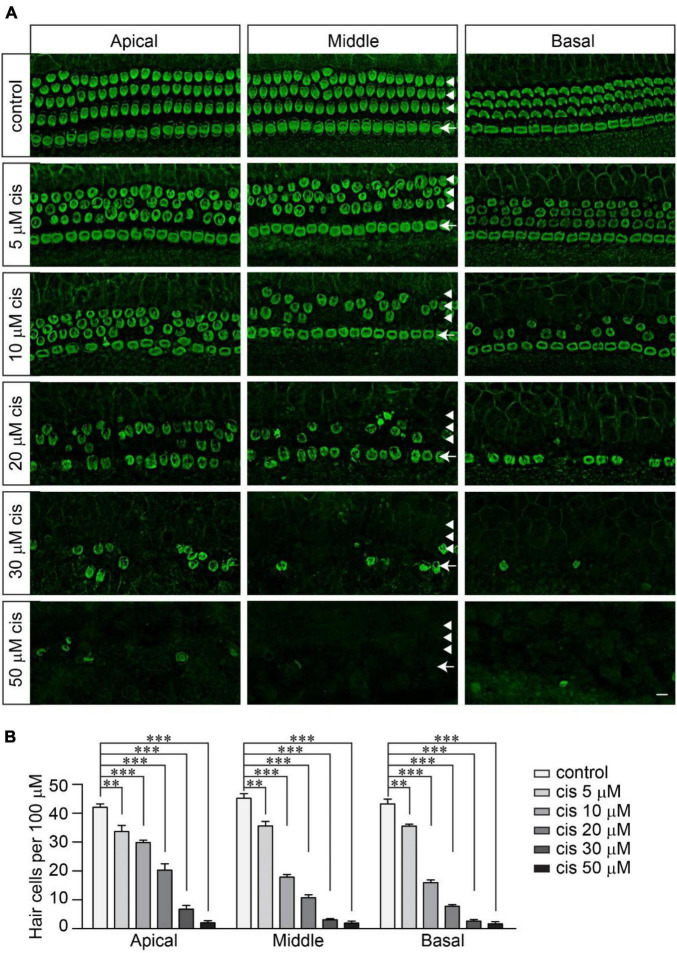
Cisplatin dose-dependently kills wild-type murine hair cells. **(A)** Cochlear explants were dissected from P3 wild-type C57/BL6J mice and cultured overnight in DMEM/F12 culture medium. Then, the cochlear explants were exposed to cisplatin at different concentrations for 2 days. The samples were fixed and labeled with an antibody against β2-spectrin, a hair cell marker. Note, OHCs (arrowheads) were more susceptible to cisplatin than IHCs (arrows). Scale bar: 10 μm. **(B)** Numbers of hair cells per 100 μm (3 mice per group). Data are represented as the mean ± standard error (SE). Two-way ANOVA followed by Bonferroni *post hoc* test was performed (^**^p < 0.01 and ^***^p < 0.001).

To investigate the extent to which functional mechanotransduction affects cisplatin ototoxicity, we first determined the concentration at which cisplatin effectively kills wild-type hair cells. Mouse cochlear explants dissected from postnatal day 3 (P3) wild-type C57/BL6J mice were cultured overnight and then exposed to cisplatin at a range of concentrations from 0 to 50 μM for 2 days. Then, the samples were fixed and labeled with an antibody against β2-spectrin, a protein highly expressed in cochlear hair cells (Holley and Ashmore, 1990; Scheffer et al., 2015; Liu et al., 2019). Without cisplatin exposure, we observed a highly organized cochlear epithelium with three rows of outer hair cells (OHCs) on the external side of the tunnel of Corti and one row of inner hair cells (IHCs) on the internal side (Figure 1A). Cisplatin caused a reliable dose-dependent loss of hair cells (Figures 1A,B). The loss of hair cells, mainly OHCs, was observed in the cochlear explants exposed to cisplatin at a concentration as low as 5 μM. Furthermore, we found that 20 μM of cisplatin led to an ∼80% reduction of hair cells in the middle region of the cochlea. Cisplatin at a 30 μM or higher concentration killed almost all of the hair cells from the apical to basal regions of the cochlea (Figures 1A,B). Consistent with previous findings (Anniko and Sobin, 1986; Cardinaal et al., 2000; Rainey et al., 2016), the OHCs were more sensitive to cisplatin ototoxicity compared to the IHCs (Figure 1A). In addition, the hair cells in the basal region were also more susceptible to cisplatin ototoxicity compared to the hair cells in the apical region (Figures 1A,B). Due to the tonotopic susceptibility of the hair cells to cisplatin toxicity, in the following studies, we imaged and analyzed the hair cells in the middle region of the cochlear explants.

### Abolishing Mechanotransduction Partially Protects Hair Cells From Cisplatin Ototoxicity

**FIGURE 2:**
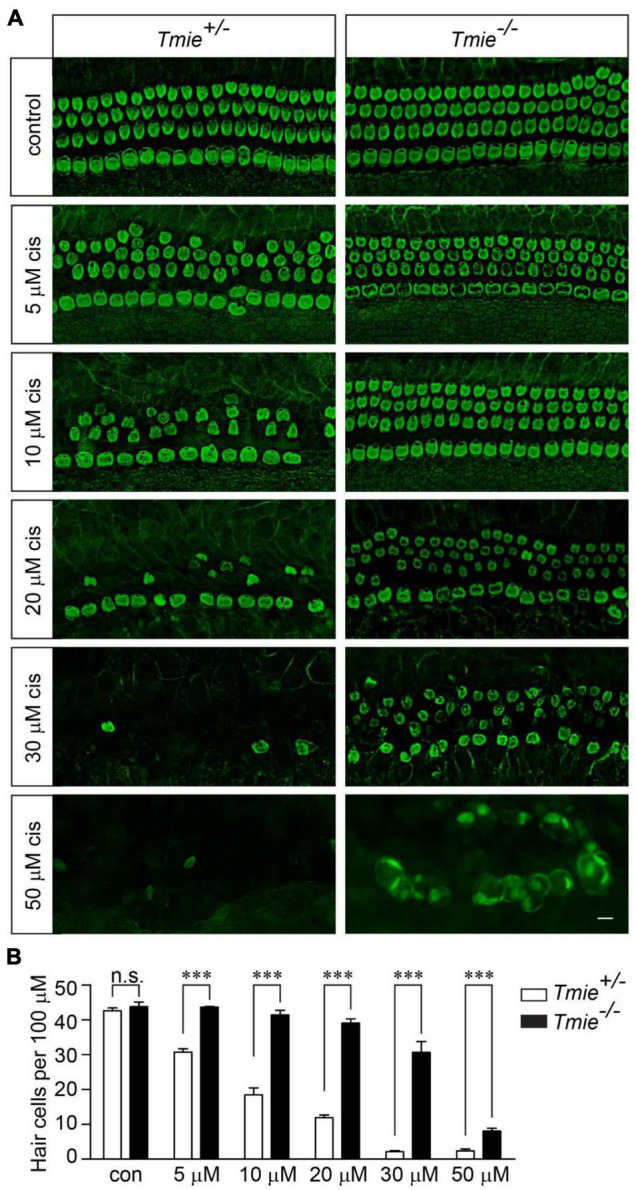
*Tmie* mutant hair cells are more resistant to cisplatin ototoxicity. **(A)** Cochlear explants were dissected from P3 *Tmie*^±^ and *Tmie*^–/–^ mice and then exposed to cisplatin at different concentrations for 2 days. The samples were fixed and labeled with an antibody against β2-spectrin. The majority of the *Tmie*^–/–^ hair cells were dead with a few swollen hair cells in the explants, after the exposure to 50 μM of cisplatin for 2 days. Note, the *Tmie*^–/–^ hair cells are more resistant to cisplatin ototoxicity. Scale bar: 10 μm. **(B)** Numbers of hair cells per 100 μm in the middle region of the cochlea (5 mice per group, control; 3 mice per group, 5 μM cisplatin; 7 mice per group, 10 μM cisplatin; 4 mice per group, 20 μM cisplatin; 4 mice per group, 30 μM cisplatin; 3 mice per group, 50 μM cisplatin). Data are represented as the mean ± SE. n.s., not significant; ^***^p < 0.001 by Student’s *t*-test.

We next sought to determine whether murine hair cells lacking mechanotransduction are more resistant to cisplatin ototoxicity. The cochlear explants were dissected from P3 *Tmie* homozygous (*Tmie*^–/–^) mice, which have no mechanotransduction current in their hair cells (Zhao et al., 2014). The samples were then exposed to various concentrations of cisplatin for 2 days. Similar to that in the wild-type hair cells, 20 μM of cisplatin killed ∼80% of the hair cells in the *Tmie* heterozygous (*Tmie*^±^) cochlear explants. Interestingly, we did not observe any significant hair cell loss in the *Tmie*^–/–^ mice after exposure to 20 μM of cisplatin (Figures 2A,B). Notably, there was significant *Tmie*^–/–^ hair cell death after exposure to 30 μM of cisplatin, but much less compared to that of the *Tmie*^±^ mice (Figures 2A,B). At concentrations of 50 μM or higher, cisplatin killed almost all of the hair cells in both the *Tmie*^±^ and *Tmie*^–/–^ (Figures 2A,B), suggesting that abolishing mechanotransduction partially protects hair cells from cisplatin ototoxicity.

### Abolishing Mechanotransduction Blocks Cisplatin Uptake

**FIGURE 3:**
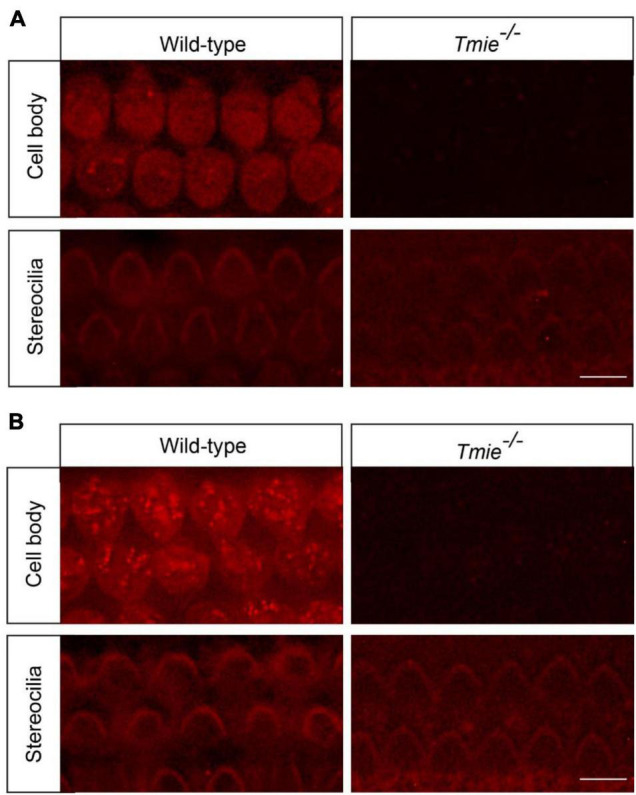
Cisplatin uptake relies on functional mechanotransduction. Cochlear explants dissected from P3 wild-type and *Tmie*^–/–^ mice were incubated with Texas Red-conjugated cisplatin (TR–cisplatin) for 15 min **(A)** or 2 h **(B)**. Note, a robust TR–cisplatin signal was detected in the cell body of the wild-type OHCs. A weak cisplatin signal was detected in the stereocilia in the wild-type and *Tmie*^–/–^ hair cells. Scale bars: 10 μm.

Impaired mechanotransduction partially protects hair cells from cisplatin ototoxicity, suggesting that functional mechanotransduction might be critical for cisplatin uptake into murine hair cells. Fluorophore–cisplatin conjugates have been widely used to study the cellular uptake of cisplatin (Thomas et al., 2013; Chu et al., 2016). Thus, wild-type and *Tmie*^–/–^ hair cells were incubated with Texas Red-conjugated cisplatin (TR–cisplatin). Fifteen minutes after the exposure to TR–cisplatin, a robust fluorescent signal was detected in the cell body of the wild-type hair cells, but not in the *Tmie*^–/–^ hair cells (Figure 3A). Two hours after the exposure to TR–cisplatin, we still did not detect a fluorescent signal in the cell body of the *Tmie*^–/–^ hair cells (Figure 3B), suggesting an essential role of mechanotransduction in cisplatin uptake. A very weak fluorescent signal at the stereociliary region was observed in the *Tmie*^–/–^ hair cells, as well as in the wild-type hair cells (Figures 3A,B).

### Otoprotective Effects of Cimetidine Do Not Require Mechanotransduction

**FIGURE 4:**
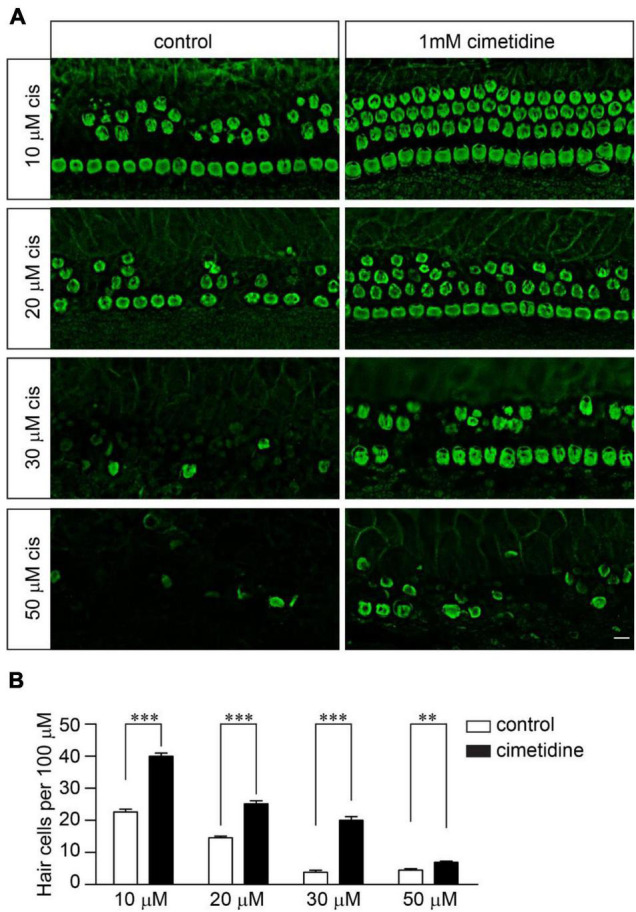
Cimetidine protects wild-type hair cells from cisplatin ototoxicity. **(A)** Cochlear explants were dissected from P3 wild-type mice and then exposed to different concentrations of cisplatin with/without 1 mM cimetidine for 2 days. The samples were then fixed and labeled with an antibody against β2-spectrin. Note, 1 mM cimetidine partially protected hair cells from cisplatin ototoxicity. Scale bar: 10 μm. **(B)** Numbers of hair cells per 100 μm in the middle region of the cochlea (5 mice per group, 10 μM cisplatin; 6 mice per group, 20 μM cisplatin; 3 mice per group, 30 μM cisplatin; 3 mice per group, 50 μM cisplatin). Data are represented as the mean ± SE. ^**^p < 0.01 and ^***^p < 0.001 by Student’s *t*-test.

**FIGURE 5:**
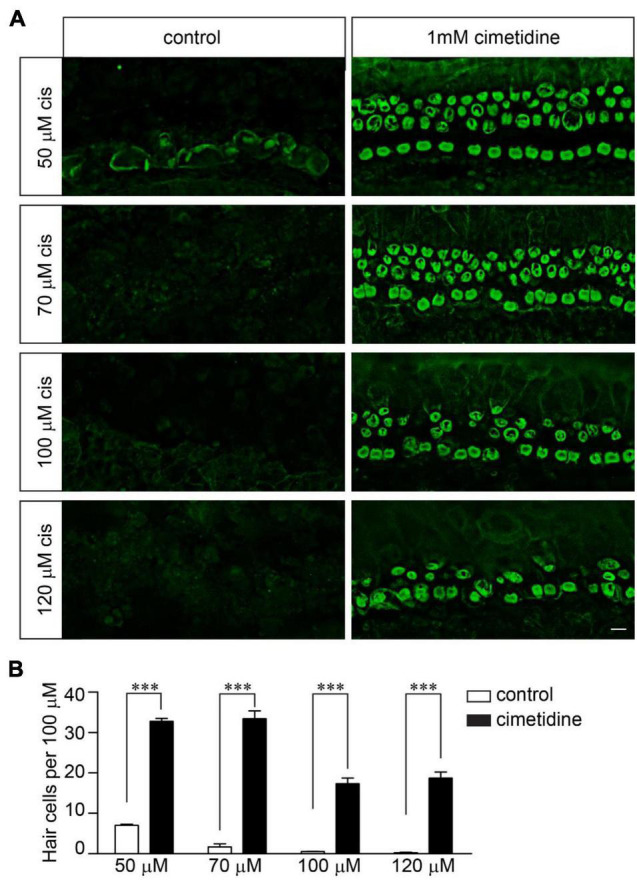
Cimetidine protects *Tmie*^–/–^ hair cells from cisplatin ototoxicity. **(A)** Cochlear explants were dissected from P3 *Tmie*^–/–^ mice and then exposed to cisplatin at the indicated doses with/without 1 mM cimetidine for 2 days. The samples were fixed and labeled with an antibody against β2-spectrin. Note, cimetidine protected the *Tmie* mutant hair cells from cisplatin ototoxicity. Scale bar: 10 μm. **(B)** Numbers of hair cells per 100 μm in the middle region of cochlea (5 mice per group, 50 μM cisplatin; 4 mice per group, 70 μM cisplatin; 3 mice per group, 100 μM cisplatin; 3 mice per group, 120 μM cisplatin). Data are represented as the mean ± SE. ^***^p < 0.001 by Student’s *t*-test.

Although OCT2 is not involved in cisplatin uptake and ototoxicity in zebrafish hair cells (Thomas et al., 2013), it might play a significant role in murine hair cells, as suggested by several studies (Ding et al., 2011). To investigate the extent to which OCT2 is involved in protecting hair cells from cisplatin ototoxicity, wild-type and *Tmie*^–/–^ hair cells were exposed to different concentrations of cisplatin with/without 1 mM cimetidine for 2 days. Cimetidine prevented hair cell death in the wild-type cochlear explants exposed to low concentrations of cisplatin (Figures 4A,B). Notably, higher concentrations of cisplatin still efficiently killed wild-type hair cells even in the presence of cimetidine (Figures 4A,B). Then, we asked whether the protection effects of cimetidine rely on mechanotransduction. Thus, *Tmie*^–/–^ hair cells were exposed to 50, 70, 100, and 120 μM of cisplatin with/without 1 mM cimetidine for 2 days. Remarkably, cimetidine also protected the *Tmie*^–/–^ hair cells from cisplatin ototoxicity (Figures 5A,B), suggesting that the otoprotective effects of cimetidine occur *via* other mechanisms instead of affecting mechanotransduction.

## Discussion

Sensorineural hearing loss occurs in 22–77% of patients treated with cisplatin (Knight et al., 2005; Kushner et al., 2006; Coradini et al., 2007; Sheth et al., 2017; Kros and Steyger, 2019). Patients suffering hearing loss nowadays benefit from the use of hearing aids and cochlear implants (Brant et al., 2021). With the upgrade in hearing aids and the improvement in cochlear implant surgery to reduce the discomfort of patients (Freni et al., 2020), outcomes will continue to improve in the future. To restore natural hearing, it will be of interest to study the molecular mechanisms underlying cisplatin ototoxicity. The uptake of cisplatin in murine hair cells is not clear. Taking advantage of *Tmie* mutant mouse, we found that genetic abolishing mechanotransduction in hair cells prevents cisplatin uptake and partially prevents cisplatin-induced hair cell death, suggesting an essential role of the functional mechanotransduction channel in cisplatin uptake and toxicity in murine hair cells. In addition, we also found that cimetidine, an OCT2 inhibitor, protects murine hair cells from cisplatin ototoxicity *via* a mechanism that does not require mechanotransduction in hair cells.

Our results show that functional mechanotransduction is critical for cisplatin uptake in murine hair cells, similar to that in the zebrafish hair cells (Thomas et al., 2013). Cisplatin at high concentrations blocks mechanotransduction current (Kimitsuki et al., 1993). The uptake kinetics of TR–cisplatin and cisplatin in hair cells might be slightly different due to their different sizes. In our studies, we did not observe a significant TR–cisplatin signal in the cell body of hair cells lacking mechanotransduction, even after a long-term incubation with TR-cisplatin (Figure 3). Consistently, abolishing mechanotransduction protects hair cells from cisplatin ototoxicity (Figure 2). These results strongly implicate the mechanotransduction channel as being a major entry route of cisplatin into murine hair cells. However, we could not exclude the possibility that cisplatin enters hair cells *via* another channel that is regulated by mechanotransduction (Thomas et al., 2013). It will be important to reconstitute the mechanotransduction machinery in a heterologous system (Zhao and Muller, 2015) and then investigate whether the mechanotransduction channel is responsible for cisplatin uptake. A very weak fluorescent signal at the stereociliary region was observed in the *Tmie*^–/–^ hair cells, as well as in wild-type hair cells (Figures 3A,B). Consistently, in zebrafish hair cells with impaired mechanotransduction, a weak cisplatin signal was also detected in the stereociliary region (Thomas et al., 2013). It is possible that some cisplatin entered hair cells *via* another route and was then retained in the stereociliary region. Another possibility is that cisplatin did not cross the cell membrane, but instead bound to the extracellular region of some stereociliary proteins. Unfortunately, the stereocilia are very tiny structures, which makes it extremely difficult to determine the detailed subcellular localization of cisplatin in stereocilia using fluorescent microscopy.

In our studies, we found that cimetidine, an inhibitor of the organic cation transporter, protected *Tmie*^–/–^ hair cells from cisplatin ototoxicity, suggesting that the otoprotective effects of cimetidine occur *via* other mechanisms instead of affecting mechanotransduction. Interestingly, previous studies found that OCT2 is highly expressed in both IHCs and OHCs in murine inner ear and mice lacking OCT1/2 are insusceptible to cisplatin ototoxicity (Ciarimboli et al., 2010). These results suggest an essential role of OCT2 in cisplatin ototoxicity. Other studies found that cimetidine could also inhibit histamine H2 receptor, which is expressed in the inner ear (Takumida et al., 2016). Thus, it is possible that histamine receptors might also be involved in cisplatin ototoxicity and characterization of mice lacking histamine receptor(s) would be informative. In our studies, we found that blocking both mechanotransduction and OCT2 did not provide complete protection against cisplatin (Figure 5), implicating the possibility of additional entry routes of cisplatin into murine hair cells. Thus, it will be of interest to investigate the extent to which other candidates such as TRP channels (Sheth et al., 2017; Jiang et al., 2019) are involved in cisplatin uptake and ototoxicity in murine hair cells.

Mechanotransduction channel is essential for auditory perception. Mutations of TMIE lead to permanent hearing loss in humans and mice (Mitchem et al., 2002; Naz et al., 2002; Zhao et al., 2014). Disrupting mechanotransduction protects hair cells from cisplatin-induced hair cell death (Figure 2). Notably, some compounds, such as d-Tubocurarine and Berbamine, could reversibly block the mechanotransduction channel, suggesting that transiently blocking the mechanotransduction channel in hair cells is a potential therapeutic method to prevent cisplatin ototoxicity (Kirkwood et al., 2017).

Besides hair cells, spiral ganglion neurons that innervate hair cells and stria vascularis cells that generate the endocochlear potential are another two major targets of cisplatin in the inner ear (Van Ruijven et al., 2004, 2005; Taukulis et al., 2021). Before entering hair cells, systemically administered cisplatin enters the stria vascularis through the blood-labyrinth barrier (Chu et al., 2016; Breglio et al., 2017; Prayuenyong et al., 2021). Revealed by LacZ staining, the expression level of *Tmie* is low in mouse stria vascularis or spiral ganglion neurons (Zhao et al., 2014). Interestingly, specific OCT2 expression was detected in the stria vascularis cells and spiral ganglion neurons (Ciarimboli et al., 2010; Hellberg et al., 2015), suggesting that OCT2 is a potential target for protecting stria vascularis cells and spiral ganglion neurons against cisplatin ototoxicity.

In summary, we found that both mechanotransduction and the organic cation transporter are critical for cisplatin ototoxicity in murine hair cells. Extensively illustrating the entry routes and ototoxic mechanisms of cisplatin in murine hair cells may lead to the development of novel therapeutic approaches to prevent cisplatin-induced hearing loss.

## Data Availability Statement

The original contributions presented in the study are included in the article/supplementary material, further inquiries can be directed to the corresponding author.

## Ethics Statement

The animal study was reviewed and approved by Institutional Animal Care and Use Committee of Indiana University School of Medicine.

## Author Contributions

JL, CL, SK, and BZ: methodology and investigation. MY and JL: counting survived cells. JL, CL, and BZ: writing manuscript. BZ: conceptualization and supervision. All authors contributed to the article and approved the submitted version.

## Conflict of Interest

The authors declare that the research was conducted in the absence of any commercial or financial relationships that could be construed as a potential conflict of interest.

## Publisher’s Note

All claims expressed in this article are solely those of the authors and do not necessarily represent those of their affiliated organizations, or those of the publisher, the editors and the reviewers. Any product that may be evaluated in this article, or claim that may be made by its manufacturer, is not guaranteed or endorsed by the publisher.
